# A Case of Dengue Fever With Hemorrhagic Manifestations

**DOI:** 10.7759/cureus.8581

**Published:** 2020-06-12

**Authors:** Muhammad Ali Raza, Muhammad Aslam Khan, Komal Ejaz, Muhammad Adnan Haider, Faisal Rasheed

**Affiliations:** 1 Internal Medicine, Conemaugh Memorial Medical Center, Johnstown, USA; 2 Internal Medicine, Allama Iqbal Medical College/Jinnah Hospital, Lahore, PAK; 3 Intrenal Medicine, The Wright Center for Graduate Medical Education, Scranton, USA; 4 Internal Medicine, Nishtar Medical University and Hospital, Multan, PAK

**Keywords:** dengue fever/complications, dhf, dengue hemorrhagic fever, mosquito-borne diseases

## Abstract

Dengue fever is an arboviral infection spread by the Aedes mosquito with a wide spectrum of presentations encompassing simple flu-like illness to hemorrhagic manifestations. Hemorrhagic complications range from simple petechiae and purpura to gastrointestinal bleeding, hematuria, and severe central nervous system (CNS) bleeds. Herein we present a case of a 38-year-old male with dengue fever along with its hemorrhagic manifestations. Additionally, we conducted an extensive review of the literature to elucidate pathophysiology, diagnosis, and management of hemorrhagic manifestations in dengue fever.

## Introduction

Dengue fever (DF) is one of the most prevalent mosquito-borne viral infections affecting humans, with multiple outbreaks recorded every year. Dengue virus (DENV) belongs to the Flaviviridae family, which is a single-stranded positive-sense RNA virus. DENV has four strains (DENV 1-4), all of which are spread by Aedes mosquito. Although most of the infections are self-limiting and asymptomatic, DENV can lead to grave complications, such as dengue hemorrhagic fever (DHF) and dengue shock syndrome (DSS). Moreover, certain serious complications, such as myocarditis, encephalopathy, liver failure, splenic rupture, acute kidney injury, pancreatitis, and muscle hematoma, can also be associated with dengue infection [1-7]. Although these complications are rare, timely diagnosis can prevent the development of these lethal complications.

## Case presentation

**Figure 1:**
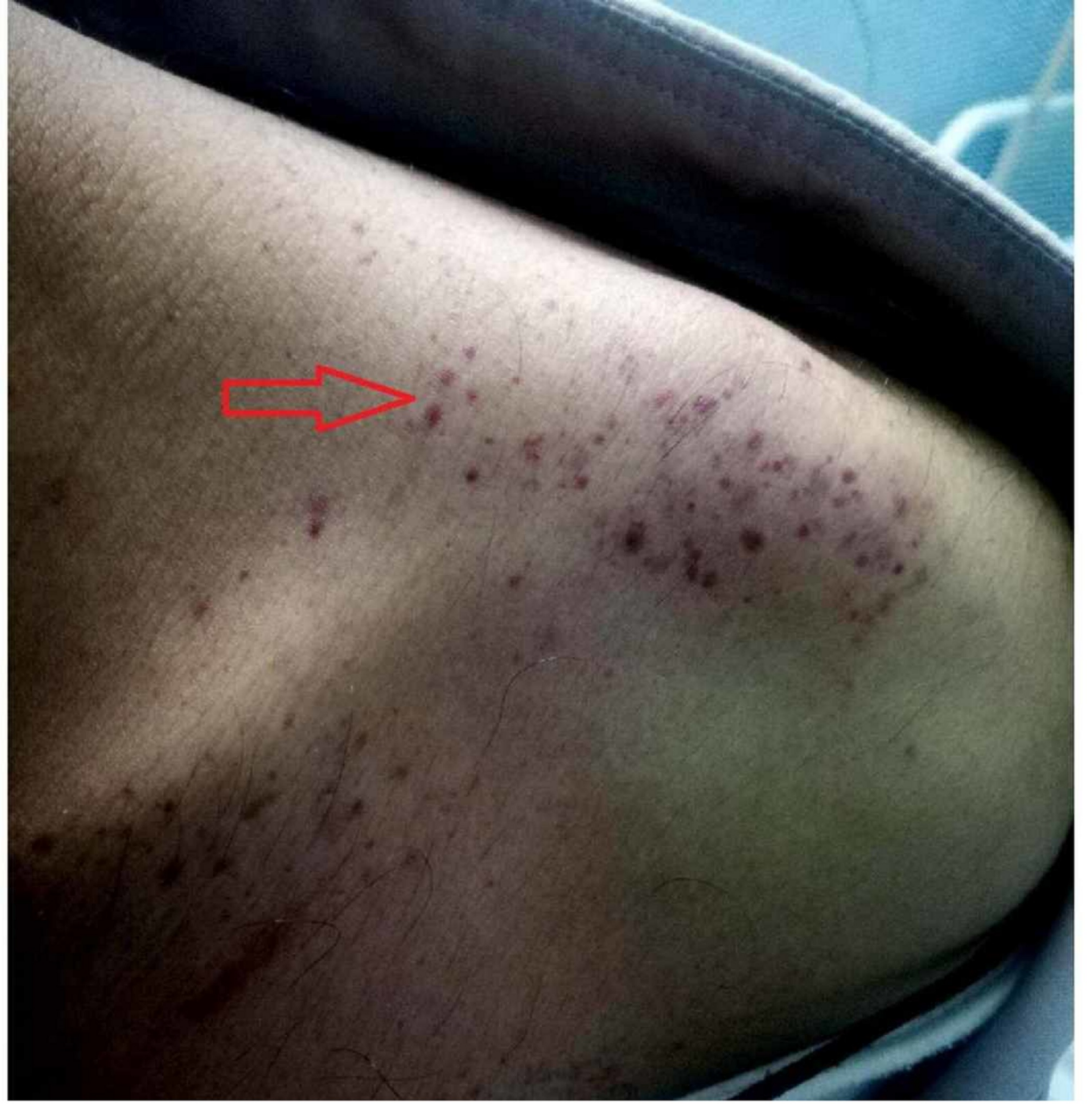
Multiple purpura and petechiae on the left shoulder

**Figure 2:**
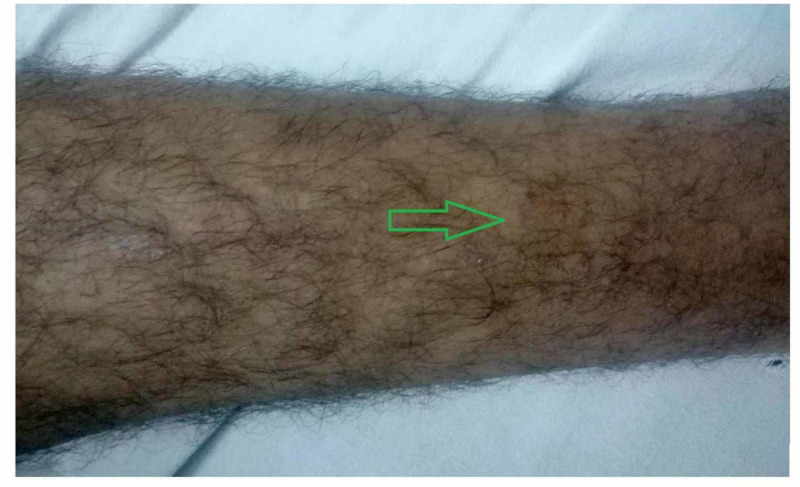
Multiple petechiae on leg

**Figure 3:**
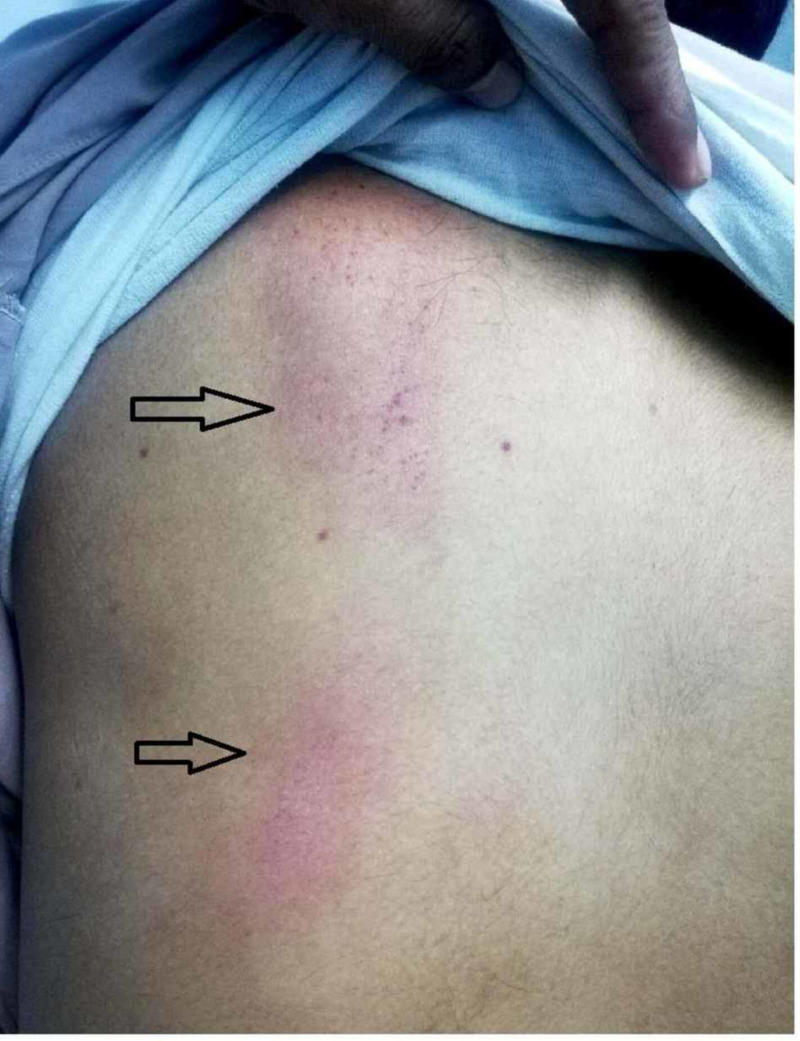
Two large ecchymotic lesions on the patient's back

A 38-year-old male with no significant past medical or surgical history presented to the emergency department (ED) with complaints of fever, headache, retro-orbital pain, and myalgia of one-week duration. The patient reported high-grade intermittent fever for the past seven days that was associated with rigors and chills. His other complaints included reddish-colored urine with clots for two days along with two episodes of gum bleed. The pertinent denials included sore throat, chest pain, shortness of breath, vomiting, abdominal pain, diarrhea, burning micturition, dysuria, urinary frequency, and contact with animals. On physical examination, the patient was vitally stable, oriented to person, place and time, and had mild conjunctival pallor. On skin examination, there were multiple purpura and petechiae on the left shoulder (Figure 1), trunk, and both legs (Figure 2), and two large ecchymotic lesions on the patient's back (Figure 3). The rest of the systemic examination was unremarkable.

The baseline workup done in the ED was normal except for a low platelet count of 20,000 x 10^9^/L and a low white blood cell (WBC) count of 2.4 x 10^9^/L. The patient was admitted in the isolation ward with the suspicion of DHF which was later confirmed on serological evidence with positive dengue-specific IgM with a value of 1.94 on enzyme-linked immunosorbent assay (ELISA). Furthermore, the tourniquet test was performed, which turned out to be positive. The patient was treated conservatively with acetaminophen one gram three times a day and one liter infusion of normal saline twice daily. The patient continued to have reddish-colored urine and had one more episode of gums bleed on his third day of admission with a platelet count of 6,000 x 10^9^/L. Infectious diseases department was consulted who recommended transfusion six units of platelets. Platelet count continued to deteriorate further with a count of 2,000 x 10^9^/L on his sixth day of admission but with clinical improvement in his fever and bleeding manifestations. The patient was monitored daily with blood counts and bleeding manifestations. The patient improved clinically on the 11th day of his admission with no hemorrhagic manifestations and normalization of blood counts (platelet count 112 x 10^9^/L and WBC count 6.3 x 10^9^/L). The patient was discharged home with a follow-up appointment one month later.

## Discussion

DF is a mosquito-borne tropical infection with an increasing number of cases each year. During the 1950s, the average number of cases reported annually to the World Health Organization (WHO) was around 900 from 10 countries. With time, the incidence of DF increased significantly with around 0.5 million cases reported in the year 2000 and 3.3 million cases recorded in the year 2015. According to an estimate, the annual incidence of DF is around 390 million with 96 million developing clinical symptoms of varying severity [8,9]. DF has variable clinical manifestations and usually presents with abrupt onset high-grade fever, myalgias, arthralgia, headache, and retro-orbital pain. A small proportion of patients progress to DHF, one of the most lethal complications of DENV infection. DHF presents with similar clinical manifestation during its febrile phase as DF initially, but it is followed by the plasma leakage phase. The febrile period usually lasts between two and seven days. The bleeding manifestations of DHF usually commence during the defervescence phase [8,9].

DHF is classified into four grades depending on the severity of clinical manifestations. (a) Grade I: positive tourniquet test without any bleeding. (b) Grade II: clinical evidence of bleeding such as ecchymosis, epistaxis, gastrointestinal hemorrhage, hematuria, menorrhagia, or rarely, bleeding into internal organs like central nervous system (CNS) and lungs. (c) Grade III: hypotension with rapid, weak pulses causing circulatory failure. (d) Grade IV presents with shock [10,11]. DHF grades III and IV are also known as DSS. The patient in our report had grade II DHF because he had ecchymosis, gum bleeds, and hematuria without progression to shock.

The precise mechanism of bleeding manifestations in DHF is unknown, but it appears to be multifactorial. Plasma leakage is the hallmark in the pathogenesis of bleeding manifestations in DHF. Several studies have proposed autoimmunity of viral infection against human cells resulting from excessive production of cytokines and chemokines, including C3a, C5a, tumor necrosis factor (TNF)-α, interleukin (IL)-2, IL-4, IL-6, IL-8, IL-10, interferon (INF)-γ, monocyte chemotactic protein (MCP)-1, and histamine. Activation of these inflammatory mediators disrupts endothelium leading to increased vascular permeability, leucopenia, thrombocytopenia, activation of the coagulation cascade, and fibrinolysis. Activation of all these cascades causes the hemorrhagic manifestations of DHF and more significantly DSS [3,9,11]. One study underscored the role of platelet-activating factor (PAF) in the pathogenesis of DHF via activation of endothelial cells and increased vascular permeability. This was further supported by measuring PAF levels in DENV infection during its different phases, which revealed higher levels of PAF during the hemorrhagic phase as compared to the febrile phase [12]. The risk of developing DHF is significantly increased in patients having a recurrent infection from DENV strain different from the one causing primary infection. The most likely mechanism is that the antibodies formed against the DENV serotype during primary infection enhance viral uptake and replication of the new serotype instead of suppressing it [8].

The management of DHF involves its accurate diagnosis and earlier detection of its hemorrhagic manifestations. For the accurate diagnosis of DENV infection, the clinical and the laboratory criteria by the WHO need to be met. The WHO clinical criteria for DHF is characterized by the following four clinical manifestations: (1) high-grade continuous fever for two to seven days; (2) a hemorrhagic tendency, such as a positive tourniquet test, or clinical signs of bleeding; (3) laboratory criteria involve thrombocytopenia (platelet less than 100,000 cells/mm^3^); and (4) hemoconcentration (a hematocrit increase of more than 20% from baseline), or pleural effusion [2,11]. There are two ways to confirm a DENV infection. Serological testing to detect antibodies against dengue virus through the ELISA assay (IgM, IgG) is considered as both sensitive and specific for primary and secondary DENV infection [2,9]. Dengue virus can also be isolated from the blood through PCR [2].

The mainstay of management of DHF/DSS is to restore intravascular volume. There needs to be close monitoring of vital signs and signs of bleeding to provide timely management. Decreased oral intake can also contribute to volume depletion in DHF. In case of compensated shock (reduced tissues perfusion signs with normal systolic blood pressure), the patient should be given intravenous (IV) fluid along with hourly monitoring of response to therapy and repeated hematocrit measurement [2,8,10]. Patients with DSS require aggressive treatment and emergent measures. Such patients should be shifted to the intensive care unit (ICU) and IV crystalloid fluid should be initiated as early as possible. Monitoring for alertness, blood pressure, heart rate, respiratory rate, oxygen saturation, and urine output should be done every 15 minutes [2,8,10]. The role of fresh-frozen plasma (FFP) and platelet transfusion is not fully established and needs to be reserved only for active bleeding and invasive procedures [1]. Platelets should be transfused only when levels drop significantly, i.e. less than 20,000 without hemorrhage or 21,000-40,000 with hemorrhage [13].

## Conclusions

DHF is one of the fatal complications of dengue virus infection, which can ultimately progress to DSS. Early recognition via stringent monitoring of vital signs and bleeding manifestations can deter the occurrence of this dreaded complication. Further research is warranted in understanding the role of platelets and FFP in the management of DHF. 
